# Exploring the challenges to using telecardiology as perceived by pre-hospital emergency care personnel: a qualitative study

**DOI:** 10.1186/s12873-023-00913-8

**Published:** 2023-12-04

**Authors:** Mostafa Bijani, Saeed Abedi, Azam Zare, Ziba Tavacol, Fozieh Abadi, Abdulhakim Alkamel

**Affiliations:** 1 Department of Medical Surgical Nursing, School of Nursing, Fasa University of Medical Sciences, Fasa, Iran; 2 Department of Emergency Medical Services, Fasa University of Medical Sciences, Fasa, Iran; 3 Department of Cardiology, Fasa University of Medical Sciences, Fasa, Iran

**Keywords:** Telehealth, Telecardiology, Emergency medical services, Qualitative research

## Abstract

**Background:**

Today, using the medical technology of telecardiology, as part of advanced medical services, plays an essential role in providing care to cardiac patients in life-threatening conditions who need emergency care. However, pre-hospital emergency care personnel are faced with certain challenges in using telecardiology, with adverse effects on their performance. Therefore, the present study aimed to investigate the challenges to using telecardiology as viewed by pre-hospital emergency care personnel in Southern Iran.

**Methods:**

The present study is a qualitative work of research with a content analysis approach. Selected using purposeful sampling, 19 pre-hospital emergency care personnel were interviewed on a semi-structured, personal, in-depth basis. The qualitative data obtained were analyzed using the Graneheim and Lundman’s conventional content analysis approach (2004).

**Results:**

Based on the qualitative data analysis, 3 themes and 8 subthemes were obtained. The three main themes included professional barriers (lack of clinical knowledge of telecardiology, lack of clinical skill in telecardiology, violation of patients’ privacy, lack of clinical guidelines on telecardiology), medical equipment and telecommunication barriers (poor reception and ineffective means of communication, low charge on the battery of tele-electrocardiogram machines), and organizational management barriers (serious lack of cardiologists available for medical counseling and lack of continual personal development of the telecardiology personnel).

**Conclusion:**

Senior managers in pre-hospital emergency care services are recommended to use the results of this study to identify the influential factors in using telecardiology and take the necessary measures to eliminate the existing barriers toward making optimal use of telemedicine, thereby improving the quality of care provided for cardiac patients.

**Supplementary Information:**

The online version contains supplementary material available at 10.1186/s12873-023-00913-8.

## Introduction

Today, high-quality pre-hospital emergency care services are an integral part of the care provided to the patients who need immediate medical attention in most societies [1]. Quality pre-hospital emergency care services, which allow for timely access to safe care [2], are an effective and efficient system for minimizing the consequences and death tolls caused by diseases and accidents [3]. Emergency medical services were created to provide pre-hospital care as a quick response to individuals’ need for treatment, keeping patients alive, and transferring the patients to hospitals, if necessary [4]. As one of the main challenges in the healthcare systems of different societies, cardiovascular diseases are the main cause of health-related fatality and disability, imposing huge costs on the healthcare systems [5, 6]. A large proportion of the missions of the pre-hospital emergency care personnel consist of providing care to cardiac patients [7]. One of the influential factors in clinical diagnosis of cardiac conditions, especially sudden cardiac arrest, is the use of medical equipment, including telecardiology, in pre-hospital emergency care services [8]. Telecardiology is an advanced method which allows for diagnosing cardiac diseases from a distance. In telecardiology, upon arriving at the cardiac patient’s side, the pre-hospital emergency care personnel attach the leads of the electrocardiogram machine and send the patient’s cardiogram to a cardiologist via an application. Thus, they can make the best clinical decision in the shortest time [9]. According to a study by Sharifikia et al. (2022), use of telemedicine in emergency care services reduces the treatment costs, improves the quality of care, reduces the patient’s treatment time, and lowers the fatality rates [10].

Despite the increased use of telecardiology in pre-hospital and hospital emergency care services, there are certain challenges and barriers to using this medical technology. Aslani et al.’s study [11] revealed that the effectiveness of telecardiology can be undermined by lack of the needed infrastructure in telecommunication, paramedics’ inadequate knowledge of telecardiology, and unavailability of a cardiologist at the time of medical counsel.

There is little knowledge about telecardiology as performed by the pre-hospital emergency care personnel and the influential factors and challenges impacting their real performance. In addition, a review of literature showed that only a few studies qualitatively investigated the use of telecardiology by pre-hospital emergency care personnel and the factors which have an impact on it in real environments in pre-hospital emergency care. In order to understand a concept and determine its dimensions, we recommend that a qualitative approach can prove useful since such studies explore a concept in its cultural context from the perspective of individuals who have have been involved with the concept for a long time [12, 13]. Without understanding the pre-hospital emergency care personnel’s experiences of using telecardiology, it is not possible to identify the obstacles and suggest effective solutions. Accordingly, the present study was conducted using a qualitative approach to explore the challenges to using telecardiology as perceived by pre-hospital emergency care personnel.

## Methods

The present study is a qualitative work of research with a content analysis approach. Conventional content analysis is usually appropriate when the existing knowledge or research literature on a phenomenon is limited [14].

### Study participants and data collection

The participants were 19 pre-hospital emergency care personnel who were selected using purposeful sampling. Nineteen Semi-structured, individual interviews were used to collect the data from September 2022 to February 2023. Each interview continued for 45 to 60 min. All the interviews were conducted by the first author (MB) in the meeting room of Fasa Emergency Medical Center, Fars province, southern Iran, with prior arrangements with the head of the emergency department and the participants. In qualitative studies, semi-structured interview is often used. It involves asking questions in a certain thematic framework. This method is basically a dialogue between the researcher and participants, directed by a flexible interview manual which is enriched by follow-up questions [14].

### Development of interview guideline

The interview guide was designed based on the research team’s views and the available literature. We also assessed the face and content validity of the interview questions using the views of 10 emergency care staff members and 5 pre-hospital emergency care experts and professors. Accordingly, Accordingly, the face validity of the interview guide was determined based on the results of three initial interviews with the participants and some minor changes were made to make the questions more comprehensible. We excluded the results of those three interviews from the final analysis. The inclusion criteria included willingness to participate in the study, at least one year of experience of practice in pre-hospital emergency care services, and experience in using telecardiology. The participants unwilling to participate in the study or withdrew from the study for any reason were excluded. Of the 30 pre-hospital emergency personnel invited to take part in the study, 11 did not accept to do so because of their busy schedules, long work shifts, and their families. Therefore, the participants were 19 pre-hospital emergency care personnel who were selected using purposeful sampling. Upon consultation with the director of the pre-hospital emergency care office, we chose one of the personnel who could help us gather rich information for the first interview. Then, he was asked to name a colleague with adequate knowledge and experience of the subject under investigation. Thus, we selected the subsequent participants who had a good understanding of the subject of the study based on the information which was provided by the participants selected earlier. Sampling continued until data saturation, when no more new data could be extracted, and no new categories or sub-categories emerged [15]. In the present study, data were saturated after 19 interviews; however, two more interviews were done to make sure no new data could be extracted. The interviews started with some general questions such as “What is your work experience?” and “Can you describe one of your workdays when you used telecardiology?”, and then followed by more specific questions based on the study objectives. These questions were “In your experience, what are the challenges with which pre-hospital emergency care personnel are faced when using telecardiology?”, “How can telecardiology be better used by the personnel?”, and “What are the skills needed for the pre-hospital emergency care personnel when using a telecardiogram machine?” All the interviews focused on the main objective of the study. In addition, follow-up questions were asked to further clarify the information provided by the participants: “Can you elaborate on the subject?”, “What do you mean by that?’ and “Can you give an example or describe one of your experiences?” Supplementary file 1: (Interview Guide and English language Interview).

### Data analysis

Using Graneheim and Lundman’s approach to content analysis (2004) [16], the researchers first read and reread the transcript of each interview to immerse in the data and obtain a general idea of the concept under study. Next, the words, sentences, and paragraphs which were significant as to major barriers to using telecardiology in pre-hospital emergency care services were determined as meaning units. The meaning units with similar ideas were categorized and labeled, and the transcripts were coded. Then, the codes were examined again and compared; similar codes merged, and the codes and transcripts were reviewed. Using similarities and differences, we classified the data and developed the categories. After in-depth reflection on the categories, we could identify the themes. Immediately after each interview, the main author (MB) transcribed the content and highlighted the significant paragraphs. We assigned a code for each meaning unit. Subsequently, the third author (AZ) checked the transcripts and confirmed the meaning units and codes. The homogeneous codes were merged; then, the categories developed. To determine the reliability of the codes, the researchers examined the categories again and compared them to the data. At last, several meetings were held, and the research team examined the codes, compared them to each other, and extracted the themes. MAXQDA v. 2007 was used to analyze the collected data. Table 1 shows an example of the coding and development of the categories and themes.

**Table 1:** An example of coding and development of categories and themes

Meaning units	Coding	Subthemes	Themes
*“On several occasions, I’ve seen some of my colleagues get confused when they want to use telecardiogram and don’t have the skills to operate the device properly. Even some of the personnel who are familiar with telemedicine have difficulty using the device. Knowledge of telemedicine alone is not enough, and you need to have the necessary skills as well. If the emergency care personnel can’t use their knowledge in practice and use their skills, they will not be competent enough.” (participant 15)*	Lack of clinical skill in using the tele-electrocardiogram Lack of skill in interpreting ECG	Lack of clinical skill in telecardiology	Professional barriers
*“Sometimes, we are dispatched to help a patient with a cardiac issue in the early hours, like three o’clock in the morning; after cardiac monitoring, we send the patient’s electrocardiogram to a cardiologist to get counsel, but, unfortunately, no one is available, and we get no counsel. This is a real issue, and we can’t know if we should administer anticoagulants, such as Plavix and aspirin, or not.” (participant 7)*	Non-availability of cardiologist for consultation	Serious lack of cardiologists available for medical counseling	Organizational management barriers

### Rigor

Lincoln and Guba’s criteria were used to confirm the trustworthiness [17]. Therefore, the researchers performed prolonged engagement with the data, member checking, and peer debriefing to verify the credibility. As to member-checking, we provided four members of the emergency medical services personnel with a copy of the coded interviews and asked them to confirm the accuracy of the data. For peer-checking, five experts were asked to monitor the process of data analysis and validate the codes and categories. The dependability of the results was enhanced using a detailed description of the methods which were used to code the concepts and themes; as also, textual and audio data were provided. Furthermore, to resolve any disagreements, two of the researchers examined the results individually and then discussed them. As to confirmability, the researchers showed the coded data to the interviewees and asked them to check the accuracy of the categories and subcategories obtained. To ensure the transferability of the study results, we provided detailed descriptions of the characteristics of the interviewees in detail, the method of conducting the interviews, the data analysis method, as well as examples of the participants’ quotes. Upon their collection, the data were analyzed to help the researchers focus on the objective of the study.

### Ethical considerations

This study was carried out based on the principles of the revised Declaration of Helsinki, a statement of ethical principles that guides the medical researchers investigating human subjects. Before the interviews, the objectives of the study were explained for all the subjects, and we ensured them about their voluntary participation in the study; moreover, the methods of collecting data, the reasons for recording the interviews, the role of the interviewer and the participants, and confidentiality and anonymity of the information were explained. Then, they signed the informed consent form in case they were willing to participate. The participants were ensured that they could withdraw from the study at any time. Moreover, the study was approved by the Institutional Research Ethics Committee of Fasa University of Medical Sciences, Fasa, Iran (ethical code: IR.FUMS.REC.1401.157).

## Results

The mean age of the participants and their work experience were 35.94 ± 4.96 and11.57 ± 3.91 years, respectively. Table 2 displays the other demographic characteristics of the participants. Three themes and 8 subthemes emerged after analysis of the qualitative data. The three main themes included professional barriers, medical equipment and telecommunication barriers, and organizational management barriers (Table 3).

**Table 2:** Individual characteristics of the participants

*Participants*	*Educational level*	*Work experience (years)*
P1	Bachelor’s degree in EMS	9
P2	Bachelor’s degree in EMS	11
P3	Bachelor’s degree in EMS	5
P4	Bachelor’s degree in EMS	7
P5	Associate’s degree in EMS	15
P6	Associate’s degree in EMS	16
P7	Bachelor’s degree in nursing	6
98	Associate’s degree in EMS	10
P9	Bachelor’s degree in EMS	19
P10	Master’s degree in nursing	10
P11	Bachelor’s degree in EMS	13
P12	Bachelor’s degree in nursing	11
P13	Bachelor’s degree in nursing	8
P14	Bachelor’s degree in EMS	14
P15	Master’s degree in nursing	6
P16	Bachelor’s degree in nursing	17
P17	Bachelor’s degree in EMS	12
P18	Bachelor’s degree in nursing	10
P19	Bachelor’s degree in EMS	13

**Table 3:** Themes and subthemes extracted from content analysis

Themes	Subthemes
Professional barriers	• Lack of clinical knowledge of telecardiology • Lack of clinical skill in telecardiology • Violation of patient privacy • Lack of clinical guidelines on telecardiology
Medical equipment and telecommunication barriers	• Poor reception and ineffective means of communication • Low charge on the battery of tele-electrocardiogram machines
Organizational management barriers	• Serious lack of cardiologists available for medical counseling • Absence of continuing personal development of the personnel in telecardiology

### Professional barriers

As one of the main challenges in using telecardiology considered by the participants, professional barriers comprised lack of clinical knowledge, lack of clinical skill, violation of patient privacy, and lack of clinical guidelines.

#### Lack of clinical knowledge of telecardiology

From the participants’ viewpoints, insufficient clinical knowledge of telecardiology is the major obstacle to using this branch of telemedicine in pre-hospital emergency care services. The participants maintained that emergency care personnel should have adequate knowledge of the principles of telecardiology, including knowledge of cardiac monitoring, ECG interpretation, and management of cardiac arrest patients. The participants recommended that the senior managers in emergency care services should take useful measures to empower the personnel and promote their knowledge in the field of telecardiology. According to one of the participants:

*“Unfortunately, no effective efforts have been made to help the personnel development their knowledge in using modern medical technologies, such as telemedicine. How am I, as a paramedic, supposed to use telemedicine without any prior knowledge or training in this area?”* (participant 9).

Another participant stated that:

*“Some of my colleagues have little clinical knowledge of cardiac monitoring or reading cardiograms or the way to manage a patient in sudden cardiac arrest; their knowledge is not up to date. Because of their poor clinical knowledge, they cannot use telecardiogram machines when they are transferring a cardiac patient.”* (participant 4).

#### Lack of clinical skill in telecardiology

Having skill in using telecardiogram machines is essential to providing effective and high-quality cardiac care in pre-hospital emergency services. The participants mentioned that, in addition to knowledge of telecardiology, paramedics should be skilled at utilizing specialized medical equipment. The ability to use telemedicine is among the most important clinical skills for emergency care personnel; they should be competent in using the medical equipment used in telemedicine.

According to one of the participants:

*“On several occasions, I’ve seen some of my colleagues get confused when they want to use telecardiogram and don’t have the skills to operate the device properly. Even some of the personnel who are familiar with telemedicine have difficulty using the device. Knowledge of telemedicine alone is not enough, and you need to have the necessary skills as well. If the emergency care personnel can’t use their knowledge in practice and use their skills, they will not be competent enough.”* (participant 15)

Another participant stated that:

*“In pre-hospital emergency services, you need to be competent in doing a lot of tasks: you must be skilled at using advanced equipment and new medical technologies to help your patients in critical conditions. Paramedics with poor clinical skills in using medical equipment are just not fit for work in pre-hospital emergency services at all.”* (participant 11)

#### Violation of patient privacy

From the participants’ perspective, another major barrier to using telecardiogram machines was violation of patient privacy. As stated by them, one of the serious issues in the field of emergency medicine in Iran is lack of female paramedics, which makes it hard to preserve patient privacy in medical emergencies when the patient is a female.

One of the participants stated that:

*“Cardiac monitoring of female patients is an issue for the paramedics; some female patients do not allow the male personnel to attach the leads of the tele-elecrtocardiogram machine to their chests, and since there are no female paramedics in pre-hospital emergency services, this issue happens again and again, making both the patients and the personnel embarrassed.”* (participant 17)

According to another participant:

*“A young lady had a sharp pain in her chest and cold sweat. Based on the history I got from her, I found she was likely to have a sudden cardiac arrest. When I wanted to perform cardiac monitoring, she didn’t let me and asked why there was not a female paramedic with us. Thus, I just had to transfer her to the hospital without any cardiac monitoring.”* (participant 6)

#### Lack of clinical guidelines on telecardiology

As another subtheme of professional barriers to using telecardiology, lack of clear and comprehensive clinical guidelines can cause confusion and uncertainty in paramedics. According to one of the participants:

*“I’ve been at a loss many times. If the cardiologist doesn’t respond to my counsel request, can I administer anticoagulants as a paramedic? The managers should issue a clear-cut set of guidelines, so that we know what to do and don’t get embarrassed and confused.”* (participant 13).

### Medical equipment and telecommunication barriers

Another main theme of the present study was barriers related to medical equipment and telecommunication, which consisted of the subthemes of poor reception, ineffective means of communication, and low charge battery of electrocardiogram machines.

#### Poor reception and ineffective means of communication

The participants’ experiences showed that the state of network coverage and radio and wireless communication in some areas was so bad that it was practically impossible to apply telecardiology. One of the participants stated that:

*“The prerequisite for using advanced medical equipment and technology is providing the infrastructure needed. When there is poor coverage in an area and nothing is done to fix it, how can we expect effective use of telecardiology in providing care to patients? There is a tele-electrocardiogram machine in the ambulance, but what is the use of it when poor reception and blind spots make it impossible for us to use it?”* (participant 19)

#### Low charge battery of tele-electrocardiogram machines

Another problem with using tele-electrocardiogram machines is inadequate charge on the battery of electrocardiogram machines. According to one of the participants:

*“One basic issue with the tele-electrocardiogram machine is that it loses charge so quickly. We’ve reported the issue to the medical equipment and IT experts at the headquarters of the emergency medical services many times, but no effective measure has been taken to deal with it. On some occasions, we couldn’t use the machine because its battery was not working.”* (participant16)

### Organizational management barriers

The theme of organizational management barriers is composed of the subthemes of lack of cardiologists for medical counseling and absence of continuing personal development of the personnel in telecardiology.

#### Serious lack of cardiologists available for medical counseling

From the participants’ point of view, one of the serious challenges to using telecardiology in pre-hospital emergency services is a severe lack of cardiologists available for medical counseling when paramedics want to use the telecardiogram machine. Without cardiologists, one of the most important links in the chain of providing care to cardiac patients via telecardiology is missing. According to one of the participants:

*“Sometimes, we are dispatched to help a patient with a cardiac issue in the early hours, like three o’clock in the morning; after cardiac monitoring, we send the patient’s electrocardiogram to a cardiologist to get counsel, but, unfortunately, no one is available, and we get no counsel. This is a real issue, and we can’t know if we should administer anticoagulants, such as Plavix and aspirin, or not.”* (participant 7)

Another participant stated that:

*“We obviously can’t use tele-electocardiograms round the clock when there is only one cardiologist available. Right now, there is just one cardiologist in this department who is expected to give counsel 24 h a day, which is practically impossible. The emergency services authorities must employ more cardiologists to increase the efficacy of the services we can give by tele-electocardiograms.”* (participant14)

#### Absence of continuing personal development of the telecardiology personnel

Absence of continuing personal development is another barrier to paramedics’ use of the electrocardiogram machine in telemedicine. Organizational managers should take necessary measures to empower the personnel in using new technologies to give care to patients. One participant mentioned that:

*“In my view, the most important thing the organizational managers should do to create the right infrastructure for making optimal use of new advanced equipment, including the tele-electrocardiogram machine, is to contribute to the professional empowerment of the personnel. Sadly, the managers here first get new equipment and technology and then remember they should’ve prepared the personnel to use the equipment first, while continuing personal development of the personnel is the most important factor in improving the quality of care, especially patient safety. Though telecardiology, as a new technology, can play a crucial part in increasing the quality of healthcare; the managers’ failure to empower the personnel in using this technology can lead to its faulty use, putting the patients’ lives at risk and having a negative impact on patient safety and the quality of care.”* (participant10)

## Discussion

Today, the technology of telecardiology, as a part of advanced emergency medical services, plays a critical role in providing care to cardiac patients in life-threatening conditions in the shortest possible time [18]. Nevertheless, the pre-hospital emergency care personnel are confronted with certain challenges in using the telecardiogram machine for managing cardiac patients, with an adverse effect on their performance. The findings of the present study indicated that professional barriers, medical equipment and telecommunication barriers, and organizational management barriers were the main obstacles to using the telecardiogram machine in pre-hospital emergency care services.

The theme of professional barriers was found to consist of lack of clinical knowledge, lack of clinical skill, violation of patient privacy, and lack of clinical guidelines. A study conducted by Bijani et al. [19] reveals that the most significant factor which influences clinical decision making adversely by paramedics in all conditions, especially when patients are in a life-threatening condition, is lack of professional capabilities, especially clinical knowledge and skills. Thus, Bijani et al. suggested that the senior managers in pre-hospital emergency services should continuously assess the professional capabilities of the personnel, and if they identify a weakness, they should take the necessary measures to empower the personnel in that area. A study carried out by Ftouni et al. [20] showed that lack of clinical knowledge was one of the major obstacles to using telecardiology in healthcare providers. Different barriers and challenges still hamper extensive use of telemedicine. Healthcare providers should cooperate with different stakeholders to utilize the proposed solutions. Further research and change in policies are recommended so that utilization of telemedicine could be optimized.

Another study conducted by Mohammadi et al. [21] showed that lack of specific clinical guidelines was one of the major obstacles to clinical decision making and caregiving in pre-hospital emergency services. Violation of patient privacy was another professional barrier to using the telecardiogram machine, as stated by the participants of the present study. Similarly, the results of a study by Torabi et al. [22] illustrated that one of the serious issues in the field of emergency medicine in Iran is lack of female paramedics, which makes it hard to preserve patient privacy in medical emergencies when the patient is a female, especially in obstetric emergencies and cardiac monitoring. It is, therefore, essential that the Iranian medical education system should take measures so that females can major in emergency medicine and employ them in medical emergency services after graduation.

Obstacles related to medical equipment and telecommunication issues formed another theme of the present study. According to a study by O’Sullivan and Schneider [23], one of the major challenges in employment and development of telemedicine in pre-hospital emergency services is lack of the necessary infrastructure, especially with regard to network coverage and reception.

Organizational management barriers were another limitation to using telecardiology in pre-hospital emergency services. This theme is comprised of lack of cardiologists for medical counseling and absence of continuing personal development of the personnel in telecardiology. In their study, Bijani et al. [19] found that one of the greatest challenges in making clinical decisions by the emergency services personnel, especially in high-risk emergencies such as sudden cardiac arrest, was the shortage of emergency medicine doctors for counseling. Thus, senior organizational managers are suggested to recruit more doctors to provide counseling in emergency medical services. Another major obstacle related to organizational management is lack of continual personal development of thestaff in employing telemedicine. Mohammadi et al. [24] reported that a key factor in improving the quality of caregiving in pre-hospital emergency services is continuing personal development of the staff, i.e. updating the clinical knowledge and skill of the personnel and enhancing their professional capabilities.

### Limitations

One of the serious issues in the field of emergency medicine in Iran is lack of female paramedics, Therefore, in the present study, all participants are male personnel. The present study explored the barriers to using telecardiogram machines only as perceived by pre-hospital emergency care personnel. Future research is recommended to address the perspectives of patients and managers of emergency care services. In addition, given the differences in economic and social conditions and the structure of emergency care services between Iran and other countries, the findings of the present study might not be generalizable. Therefore, it is suggested that a similar study should be conducted in other countries.

### Strengths

Few studies have qualitatively investigated the major barriers to using telecardiology by pre-hospital emergency care personnel; this study is one of them.

### Suggestions

Suggestions for eliminating the challenges to using telecardiograms in pre-hospital emergency care including; professional empowerment and promotion of the knowledge and skill of pre-hospital emergency care personnel in using specialized medical equipment and new technologies, regular assessment of the personnel’s professional capabilities, planning for promotion of the knowledge and skill of the personnel in making optimal use of new medical technologies including telemedicine, providing the necessary information technology (IT) infrastructure, e.g. proper network coverage and wireless telecommunication, availability of a cardiologist for consultation.

## Conclusion

Professional barriers, medical equipment and telecommunication barriers, and organizational management barriers are among the major obstacles to using telecardiology in pre-hospital emergency care services. The authorities and managers in pre-hospital emergency care services are suggested to use the findings of the present study to identify the factors influencing the use of telecardiology and make an attempt to eliminate the existing barriers toward making optimal use of telemedicine, thereby improving the quality of care provided to cardiac patients.

### Supplementary Information

**Additional file 1: Supplementary file 1.** Interview Guide and English Language Interview.

## Data Availability

The datasets used and/or analysed during the current study are available from the corresponding author on reasonable request.

## References

[1] Reay G, Norris JM, Alix Hayden K, Abraham J, Yokom K, Nowell L (2017). Transition in care from paramedics to emergency department nurses: a systematic review protocol. Syst Rev.

[2] Gale J, Coburn A, Pearson K, Croll Z, Shaler G (2017). Developing program performance measures for rural emergency medical services. J Prehosp Emerg Care.

[3] Howard I, Cameron P, Wallis L, Castrén M, Veronica LV (2019). Identifying quality indicators for prehospital emergency care services in the low to middle income setting: the South African perspective. Afr J Emerg Med.

[4] Aringhieri R, Bruni ME, Khodaparasti S, van Essen JT (2017). Emergency medical services and beyond: addressing new challenges through a wide literature review. Comput Oper Res.

[5] Mensah GA, Roth GA, Fuster V (2019). The global burden of cardiovascular diseases and risk factors: 2020 and beyond. J Am Coll Cardiol.

[6] Roth GA, Mensah GA, Fuster V (2020). The global burden of cardiovascular diseases and risks: a compass for global action. J Am Coll Cardiol.

[7] Alrawashdeh A, Nehme Z, Williams B (2021). Impact of emergency medical service delays on time to reperfusion and mortality in STEMI. Open Heart.

[8] Mohammadi Janbazloufar K, Pazokian M, Safari M, Saberian P, Nasiri M (2020). The impact of telecardiology on the outcome of patients with myocardial infarction transported by Tehran’s emergency medical services to selected hospitals of Tehran city. Nurs Pract Today.

[9] Molinari G, Molinari M, Di Biase M, Brunetti ND (2018). Telecardiology and its setting of application: an update. J Telemed Telecare.

[10] Sharifi Kia A, Rafizadeh M, Shahmoradi L (2022). Telemedicine in the emergency department: an overview of systematic reviews. J Public Health (Berl).

[11] Aslani N, Garavand A, Jelvay S (2022). Advantages and challenges of telecardiology and providing solutions for its successful implementation: a scoping review. Int Cardiovasc Res J.

[12] Lewis S (2015). Qualitative inquiry and research design: choosing among five approaches. Health Promot Pract.

[13] Bradshaw C, Atkinson S, Doody O (2017). Employing a qualitative description approach in health care research. Glob Qual Nurs Res.

[14] Doyle L, McCabe C, Keogh B, Brady A, McCann M (2020). An overview of the qualitative descriptive design within nursing research. J Res Nurs.

[15] Renjith V, Yesodharan R, Noronha JA, Ladd E, George A (2021). Qualitative methods in health care research. Int J Prev Med.

[16] Graneheim UH, Lundman B (2004). Qualitative content analysis in nursing research: concepts, procedures and measures to achieve trustworthiness. Nurse Educ Today.

[17] Cypress BS (2017). Rigor or reliability and validity in qualitative research: perspectives, strategies, reconceptualization, and recommendations. Dimens Crit Care Nurs.

[18] Brunetti ND, Gennaro LD, Amodio G (2010). Telecardiology improves quality of diagnosis and reduces delay to treatment in elderly patients with acute myocardial infarction and atypical presentation. Eur J Cardiovasc Prev Rehabil.

[19] Bijani M, Abedi S, Karimi S, Tehranineshat B (2021). Major challenges and barriers in clinical decision-making as perceived by emergency medical services personnel: a qualitative content analysis. BMC Emerg Med.

[20] Ftouni R, AlJardali B, Hamdanieh M (2022). Challenges of telemedicine during the COVID-19 pandemic: a systematic review. BMC Med Inform Decis Mak.

[21] Mohammadi F, Tehranineshat B, Bijani M (2021). Management of COVID-19-related challenges faced by EMS personnel: a qualitative study. BMC Emerg Med.

[22] Torabi M, Borhani F, Abbaszadeh A (2018). Experiences of pre-hospital emergency medical personnel in ethical decision-making: a qualitative study. BMC Med Ethics.

[23] O'Sullivan SF, Schneider H. Developing telemedicine in Emergency Medical Services: A low-cost solution and practical approach connecting interfaces in emergency medicine. J Med Access. 2022;6:27550834221084656. 10.1177/27550834221084656. 10.1177/27550834221084656 PMC9413501 36204523

[24] Mohammadi F, Hatami M, Rezapour-Nasrabad R (2021). Study of pre-hospital emergency care personnel’s perception of ethical and clinical caring challenges in the field: a qualitative study. Rev Latinoam De Hipertens.

