# Supplementary material for: Exploring the challenges to using telecardiology as perceived by pre-hospital emergency care personnel: a qualitative study
Source: BMC Emerg Med. 2023 Dec 4;23:143. doi: 10.1186/s12873-023-00913-8 (PMC10696672; doi:10.1186/s12873-023-00913-8)
Supplement: Supplementary file 1 — Additional file 1: Supplementary file 1. Interview Guide and English Language Interview. [file 12873_2023_913_MOESM1_ESM.doc]

**Supplementary file 1: (Interview Guide and English language Interview)**

| **Interview Guide**  Thank you for accepting to be interviewed by us. The study we are undertaking is to understand more about the Thank you for accepting to be interviewed by us. The study we are undertaking is to understand more about the “Exploring the challenges and barriers to using telecardiology as perceived by pre-hospital emergency care personnel: a qualitative study”.  I will be asking you several questions which are relevant to the study. You may respond to these queries in any way you feel comfortable. It is perfectly fine if you do not want to respond. At any point during the interview, if you are not clear about any questions, you are free to clarify the same with us and ask us to explain further. The information obtained during the interview will be kept confidential and will be shared only with the research team. We would like to audio record the interview in order to ensure that we do not miss out any salient issues. The recordings will be kept confidential. Your identity will be protected and your interview will also be labeled in codes. Is it OK with you that we audio record the interview?  **Interview questions**:  1." Can you describe one of your work days on which you used telecardiology?”  2. “In your experience, what challenges pre-hospital emergency care personnel faced with in using telecardiology?”  3. How can the personnel make better use of telecardiology?”  4. “What skills do the pre-hospital emergency care personnel need to use a telecardiology?  **English language version of interview**  Date: 7 September 2022 9. 30 Am Participant 9  Hello dear, thank you for your time and attention and participation in the interview.  Would you please explain about your experience what challenges pre-hospital emergency care personnel faced with in using telecardiology?”  Based on my experiences, The poor clinical knowledge of telecardiology is a major obstacle to using this branch of telemedicine in pre-hospital emergency care services. “Unfortunately, no effective efforts have been made to help the personal development of the personnel in using modern medical technologies, such as telemedicine. How am I, as a paramedic, supposed to use telemedicine without any prior knowledge or training in this area?”  What is your solution to solve this challenge that you mentioned?  Senior managers in emergency care services should take more effective measures toward empowering and promoting the personal development of the personnel in the field of telecardiology.  Can you give others challenges pre-hospital emergency care personnel faced with in using telecardiology?  Lack of clinical skill in telecardiology. Lack of clinical guidelines on telecardiology , Poor reception and ineffective means of communication and Absence of continuing personal development of the personnel in telecardiology. |
| --- |
